# Supplementary material for: Disjoining Pressure of Water in Nanochannels
Source: Nano Lett. 2021 Aug 30;21(18):7769–74. doi: 10.1021/acs.nanolett.1c02726 (PMC8461650; doi:10.1021/acs.nanolett.1c02726)
Supplement: Supplementary file 1 — nl1c02726_si_001.pdf [file nl1c02726_si_001.pdf]

Supporting Information:
Disjoining Pressure of Water in Nanochannels

An Zou, Sajag Poudel, Manish Gupta, and Shalabh C. Maroo*
Department of Mechanical and Aerospace Engineering, Syracuse University, Syracuse, NY 13244
*Corresponding author: scmaroo@syr.edu

S1 Sample Fabrication

The samples for experiments of water filling in nanochannels consists of two parts: nanochannels formed by bonding two wafers together; and reservoirs. The fabrication process started from a silicon (Si) wafer. ∼3 μm thick photoresist (PR) was spinning coated and patterned through a standard photolithography process. Then the wafer was etched down for a certain depth to form open nanochannels, using patterned PR as mask. After removing the remaining PR, ∼1 μm thick SiO_2 film was deposited using plasma enhanced chemical vapor deposition (PECVD), and was patterned by standard photolithography followed by dry etching, to serve as the hard mask for deep Si etching (Bosch process) for reservoirs. Then, after removing the remaining PECVD SiO_2 film using buffered oxide etchant, a new 300 nm thick PECVD SiO_2 film was deposited prior bonding. The nanochannels were closed by by bonding a glass piece above the channels using standard anodic bonding. The images from atomic force microscope (AFM) showing channel profiles in Fig. 1-b in main manuscript were taken immediately before bonding.

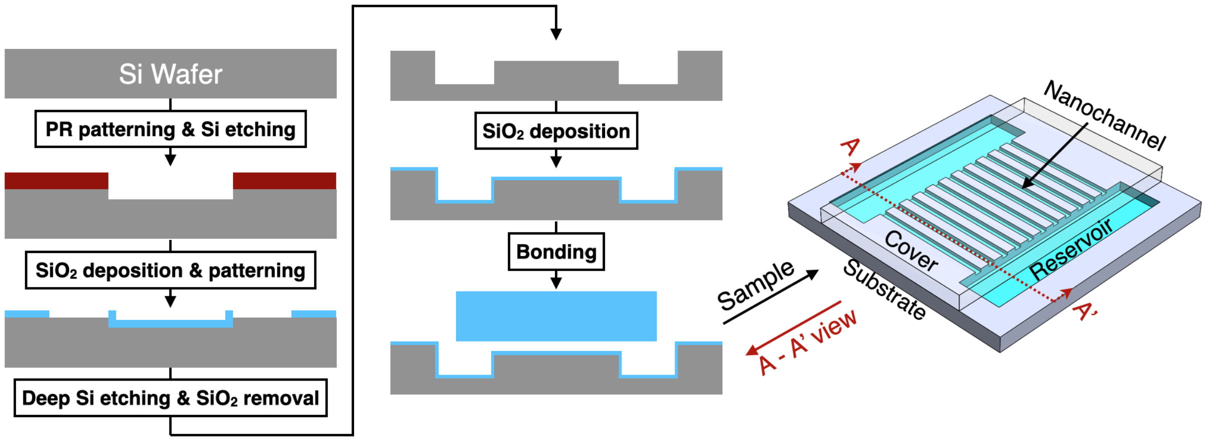

Figure S1: Nanochannel sample fabrication process.

Before conducting an experiment, the sample was cleaned using deionized (DI) water, followed by oxygen plasma cleaning for 30 min. The waiting time between plasma cleaning and experiments (ranged from a few minutes to two days) turned out not to affect the wicking rate in our experiments.

S2 Wicking Rate in Nanochannels

Although the linear dependence of filling distance on $t^{1/2}$ holds at nanoscale, the experimental filling rate is reduced from Washburn equation prediction [1, 2, 3, 4, 5]. There have been five

potential explanations for this deviation reported so far: (1) electroviscous effect [1, 6], which is a considerable increase of the fluid viscosity due to the high surface-to-volume ratio of nanochannel [7, 8]; (2) geometrical effect [9, 10]; (3) use of an intrinsic contact angle in Washburn equation [11, 4, 5, 12]. However, these effects do not explain our observed deviation. First, theoretical studies have shown as less as ∼1% contribution of electroviscous effect to reduction of the filling rate [3, 13], while it is challenging to experimentally investigate the electroviscous effect without disrupting other factors, such as disjoining pressure; what's more, experimental finding conflicts [2] with theoretical prediction of decreasing viscosity with decreasing channel height [14]. Thus, electroviscous effect itself is not enough to explain the deviation. Second, there is evidence that the top wall of the nanochannel deforms bending downward due to the significant negative pressure in the meniscus [9, 10]. The actual height (h_a) at the channel center is given by $h_a = h_0 + \alpha P$, where h_0 is the nominal height, α is a constant, and P is the absolute pressure in the fluid [2]. For a channel made by bonding a glass wafer to a silicone substrate with width in the order of 10 μm, $\alpha = 0.039\text{nm}/10^5\text{Pa}$ [2]. Estimating $P = -2\sigma \cos\theta / h_0$ (where $\theta = 0°$ for the worst scenario), the deformations of the channel at the center ($h_0 - h_a$) are 1.0 nm for 59 nm channel, 0.6 nm for 87 nm channel, 0.5 nm for 124 nm channel, and 0.06 nm for 1015 nm channel, respectively. Thus, geometry effect is negligible in current work.

S3 Dynamic Contact Angle

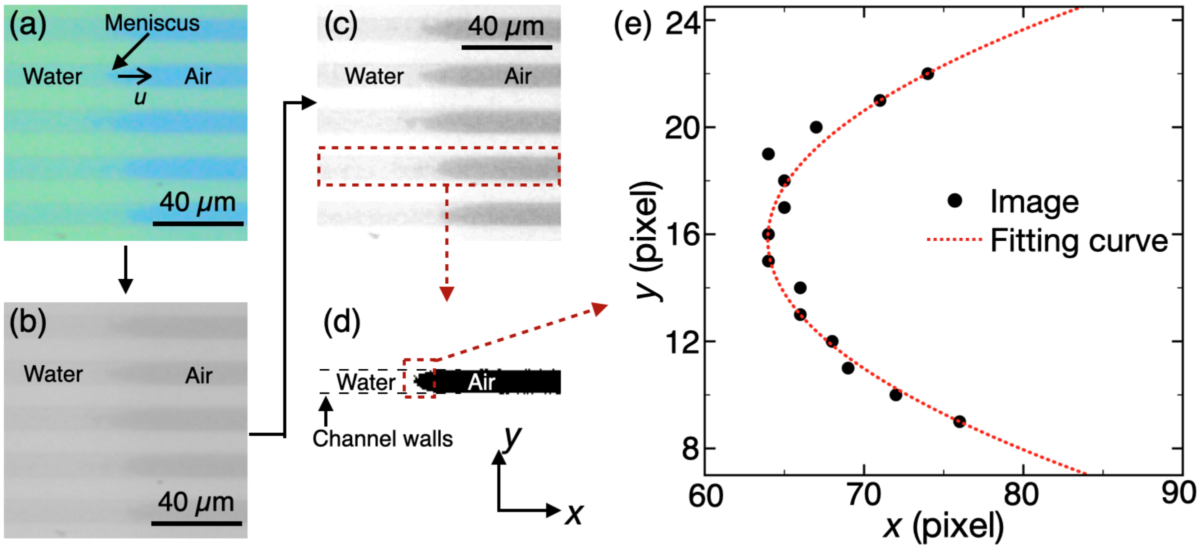

Figure S2: Contact angle calculation process from frames of the high speed videos.

The dynamic contact angles of water in nanochannels during filling were obtained from high speed videos with 1000 frames per second. Figure S2 shows the image processing process for contact angle calculation. The original image (Fig. S2-a) was first converted to gray scale (Fig. S2-b), then the image contrast was enhanced using histogram equalization (Fig. S3-c). After that, the region for the tested channel was selected to be further converted to a binary image, with a proper threshold level (Fig. S2-d). The pixels with luminance greater than the threshold were turned to value one (white); and the rest were given value of zero (black). For all images, the proper threshold level lies in the range from 0.75 to 0.90. The obtained binary image was scanned pixel by pixel to identify the location of the first black pixel in each line, which represented meniscus shape. A parabolic fitting curve for these scattered points indicated the continuous meniscus shape. To consider the apparent contact angle only, the

points in microlayer were not included in the curve fitting. For a certain point, if it was 5 pixels or more away from the adjacent point in x direction, it was treated as in the microlayer. The contact angles were then obtained from the first derivative of the parabolic fitting curve at upper and lower boundaries of the meniscus (Fig. S2-e). The filling distance was determined by the distance between entrance and the center of the meniscus.

The above process was repeated for each frame of the video to obtained the plots in Fig. 3-b in main manuscript. For a certain frame, the contact angle is the average of those at upper and lower boundaries. The data only from the frames where the R^2 value for the curve fitting was larger than 0.8 were included.

S4 MD Simulation

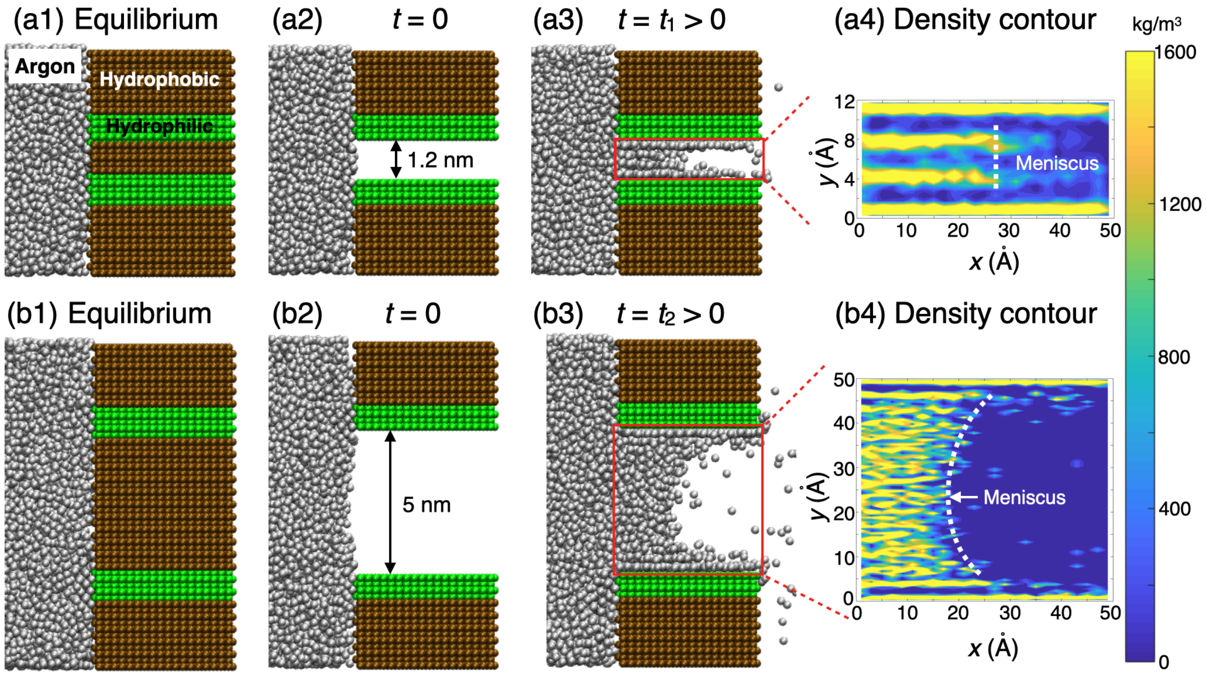

Figure S3: MD simulations for liquid filling in (a) 1.2 nm and (b) 5.0 nm channels.

In our molecular dynamics (MD) simulations, Argon fluid (silver atoms in Fig. S3) was chosen as its thermodynamic properties, obtained from MD statistically, have been well established against experimental data over the entire temperature range using basic 12-6 Lennard-Jones (L-J) potential [15]. The channel was formed between two 5 nm long parallel hydrophilic surfaces (green atoms in Fig. S3) with distance of 1.2 nm and 5.0 nm respectively. The space between hydrophilic surfaces and simulation domain boundaries were filled with hydrophobic atoms (brown ones in Fig. S3). The atomic interactions between atoms are governed by Lennard - Jones (L-J) potential ($\phi = 4\varepsilon[(\frac{\sigma}{r})^{12} - (\frac{\sigma}{r})^{6}]$) with an inner cut-off distance (r_{in}) of 1.8 nm and an outer cut-off distance (r_c) of 2.0 nm. The hydrophilic surface was mimicked by setting ε_{Ar-HL} as 1.5 times ε_{Ar-Ar} (1.67×10^{-21}); while for the hydrophobic surface, ε_{Ar-HP} was set as 1% of ε_{Ar-Ar} to have a weak interaction. All simulations were run in LAMMPS [16]. In each case, the bulk liquid was equilibrated at 90 K for 3 ns (600000 steps) in a canonical NVT ensemble (N is the number of atoms, V is the volume, and T is the temperature) using Nose-Hoover thermostat [17, 18]. The channel was filled with hydrophobic atoms to avoid any filling at this stage (Fig. S3-a1, b1). The number of atoms was tuned to make the bulk pressure close to the saturated pressure at 90 K (1.32 atm).

After reaching equilibrium, the hydrophobic atoms inside the channel were removed and the automatic filling started (Fig. S3-a2, b2). The structured liquid layer adjacent to the surface moved much faster than the bulk. In 1.2 nm channel, the solid-liquid interaction overwhelmed liquid-liquid interaction at the center height of the channel, attracting liquid atoms to move forward in parallel, and therefore showing a 90° θ_{side} (Fig. S3-a3, a4). However, in 5 nm channel, liquid-liquid interaction dominated at the channel center, thus the concave meniscus formed to minimize the surface energy (Fig. S3-b3, b4). Excluding the monolayers, and using the similar method in Section S2, the contact angle (θ_{side}) was 43.3° obtained from a parabolic curve fitting with R^2 value of 0.94.

S5 Disjoining Pressure Calculation

Equation 3 in main manuscript was used to calculate average disjoining pressure over half the channel height. The slope of the wicking rate (Fig. 2) was obtained by best linear curve fitting with highest R^2 value; θ_{top} was obtained as described in section S3. Table S1 lists the contact angles used for calculation and corresponding capillary pressure P_c and disjoining pressure P_d. It should be noted that the large deviations of θ_{top} in 59, 87, and 124 nm channels are from the fluctuation of the contact angle with time (Fig. 3b in main manuscript).

Table S1: Contact angles and corresponding capillary/disjoining pressure

Channel Height (nm)	θ_{top} (°)	θ_{side} (°)	P_c (Pa)	P_d (Pa)
59	29.4 ± 16.8	90	1.25×10^4	1.51×10^6
87	27.3 ± 13.2	90	1.28×10^4	1.29×10^6
124	40.6 ± 17.2	40.6 to 90	4.46×10^5	4.66×10^5
1015	39.6 ± 2.1	39.6 ± 2.1	1.09×10^5	1.76×10^3

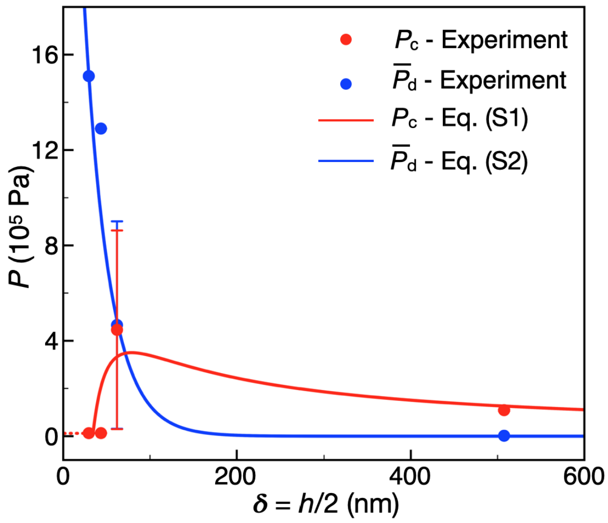

Figure S4: Capillary Pressure and disjoining pressure variation with film thickness (half of the channel height).

The contribution of the capillary pressure to wicking is highly dependent on θ_{side} due to the high aspect ratio of the channels (Eq. S1). For small channel height, $\theta_{side} = 90°$, capillary pressure only depends on the channel width w and θ_{top}. As channel height increases, the liquid structuring effect diminishes, thus θ_{side} decreases to θ_{top}, showing uniform contact angle. To

further explore the roles of capillary pressure and disjoining pressure on wicking in nanochannels, we estimated the dependence of the θ_{side} on film thickness δ (half of the channel height) using power function in the form of $\theta = A\delta^b$ with highest R^2 value. Using Eqs. S1 and S2 (Eq. 4 in the main manuscript) for estimations of capillary pressure and disjoining pressure respectively, we obtain the comparison of capillary pressure to disjoining pressure with increasing channel height, as shown in Fig. S4, capillary pressure dominates the wicking in a channel with height larger than ∼250 nm, i.e., disjoining pressure is not important in a film thicker than ∼125 nm. Here, we chose the criterion of the dominance of the capillary pressure as where the disjoining pressure is 10% of capillary pressure.

$$P_c = 2\sigma\left(\frac{\cos\theta_{side}}{h} + \frac{\cos\theta_{top}}{w}\right) \tag{S1}$$

$$\overline{P}_d = 4.25 \times 10^6 \cdot e^{-0.035\cdot\delta} \tag{S2}$$

S6 CFD Simulation of Wicking

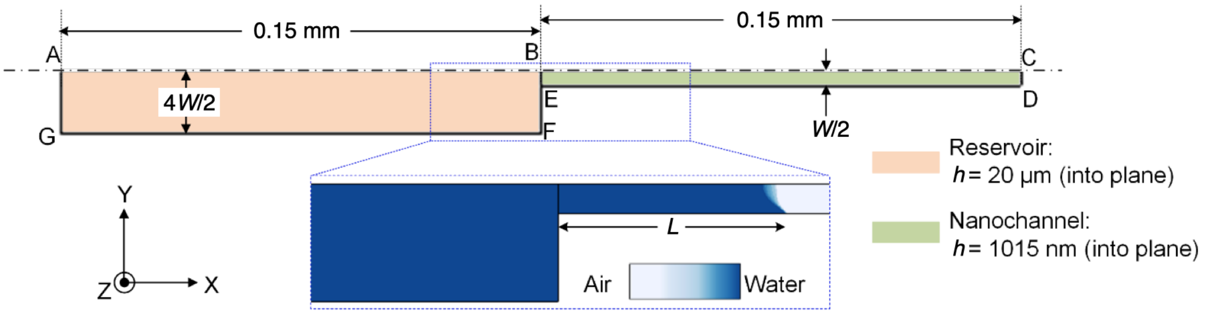

Figure S5: Top-view of the computational domain used for CFD simulations along with a snapshot of liquid propagation inside 1015 nm nanochannel at t = 100 μs.

We simulated water filling in 59 nm and 1015 nm channels respectively. In CFD simulations, the nanochannels were connected to a 20 μm high reservoir (Fig. S4). Only a one-fourth portion is considered for simulations since the domain is symmetric along the XZ plane (Fig. S5). The two zones of the computational domain: ABEFG and BCDE correspond to the reservoir and the nanochannels respectively, where liquid (water) is initially filled in the former and the characteristic of liquid filling into the latter is studied. In order to perform the numerical study, laminar multiphase simulation with volume of fluids methods is opted where the discretized form of the governing equations (continuity, momentum, energy) is solved simultaneously using finite volume methods. Liquid phase (water) is initially filled in the reservoir (ABFG) region only. No-slip boundary condition on all the solid walls and the pressure outlet condition (1 atmospheric pressure) at the exit (CD and AG) is applied. In order to establish wall adhesion for non-slip walls, the contact angle is assigned as follows: 29.3° for all the reservoir walls and side-wall of nanochannel; 29.3° for top and bottom walls in 1015 nm channel; and 90° for top and bottom walls in 59 nm channel. Capillary pressure is induced by employing continuum shear force model along with wall adhesion with specified contact angles. Further, the disjoining pressure is supplemented to the liquid phase near meniscus inside the nanochannel by using a user defined function involving ADJUST macro in ANSYS Fluent.

The filling distance evolution with time was obtained from phase contour plots, as shown in Fig. S1 for 1015 nm channel at $t = 100\mu s$. To test the grid dependence, three different sizes of mesh were created using ANSYS Workbench (Grid-1, Grid-2, and Grid-3) to have ∼31,000, ∼72,000, and ∼110,000 nodes respectively in 1015 nm channel. The results of Grid-2 are found

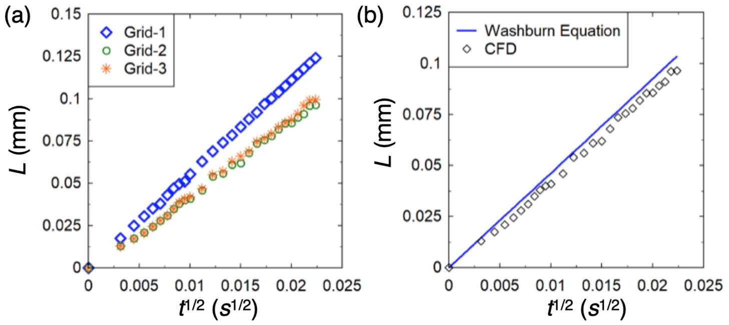

Figure S6: (a) Grid independence test; and (b) comparison of CFD result of capillary filling in a nanochannel with Washburn equation prediction in 1015 nm channel.

to be consistent with that of Grid-3 (Fig. S6-a), thus the optimum mesh (Grid-2) is chosen for the rest of the simulations in the present work. The validity of the chosen numerical methods was confirmed by a case where capillary driven filling (capillary pressure as driving force only) in 1015 nm channel was simulated, and the CFD results are in good agreement to the prediction from the Washburn equation (Eq. 2 in the main manuscript), as shown in the Fig. S6-b.

S7 Nucleation Temperature of Water in Nanochannel

S7.1 CFD Simulations of Bubble Nucleation in Nanochannels

The obtained disjoining pressure of water in SiO_2 was verified by independent investigation of nucleation temperature of water in 59 nm channel. From expression of average disjoining pressure ($\overline{P}_d$) as a function of film thickness δ (Eq. 4 in the main manuscript), we derived local disjoining pressure as a function of distance from the surface x in the form of $P_d = 5.765\exp(-0.142x)$ [19]. The derived expression of P_d was implemented in ANSYS Fluent as a user defined function, to set the local pressure at the finite volume centers (or grid points) along the height. The computational domain consists of a nanochannel of height $h = 59$ nm, width $W = 10$ μm, and length $L = 20$ μm (Fig. S7). Due to the symmetric nature of the system, only a quarter of the channel was simulated.

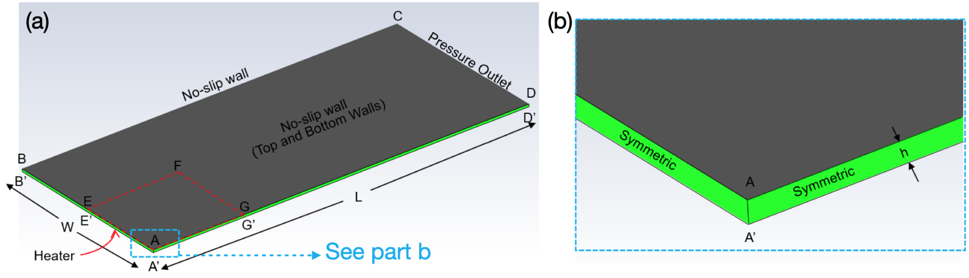

Figure S7: Simulation domain for nucleation temperature in nanochannels.

Initially, the nanochannel domain is completely filled with water at 300 K. Due to variation of P_d along the channel height, the lowest value of pressure occurs at the channel center (mid-height) as it is farthest away from the surface. Next, a constant heat flux is supplied at the specified spot of the bottom wall which raises the liquid temperature inside the domain. In the present model, phase transition (from water to vapor) is evoked using Lee Model where the coefficient of phase transition controls the rate of mass transfer and the saturation temperature is taken as the function of local pressure. Finally, bubble nucleation is observed first at the mid-height of the nanochannel where the local temperature surpasses the saturation temperature

corresponding to local pressure. Adjusting the coefficient of phase transition does not affect the nucleation temperature as it only dictates the bubble growth or decay rate after the bubble has nucleated. The applied heat flux also does not affect nucleation temperature but only changes the time instant when nucleation occurs. Thus, the temperature at which the nucleation occurs is a function of the pressure only.

S7.2 Experiments of Bubble Nucleation in Nanochannels

The bubble nucleation temperature was also measured using infrared (IR) camera equipped with 4× IR microscope (resolution: ∼3.8 μm). As shown in Fig. S8, to achieve bubble nucleation in the channel, a blue CW laser beam (wavelength of 447 ± 5 nm) was introduced into an inverted microscope, passed through a 50× objective, and focused on the sample to generate a highly localized heating area. The same objective was used for observation. In the meantime, an IR camera was used to measuring temperature from the top. Similar to the samples for wicking experiments, the nanochannel (height h ∼54 nm, width W ∼10 μm, and length L ∼200 μm) for nucleation temperature measurement is also connected to a reservoir at each end. Due to the disjoining pressure effect, we expect higher nucleation temperature in nanochannels, compared to bulk phase. During the experiments, a droplet was placed in the reservoir, and water wicked into the channel automatically. The laser power was then increased from zero incrementally until a bubble formed in the channel. The bubble stayed at steady state as the evaporation at the bubble center was balanced by the condensation occurred at the bubble ends. The bubble disappeared immediately if the laser was blocked and formed again right after the block was moved away. A video recorded by the IR camera for the steady bubble was used to obtain the nucleation temperature, which was identified as the highest temperature at each frame. The maximum temperatures from all frames were averaged as the nucleation temperature. The measurement was repeated in different channels for at least ten times to ensure repeatability. The nucleation temperature in 53 nm channel was measured as 123.9 ± 3.0 °C, which is in good agreement with CFD simulation, thus independently validating the disjoining pressure obtained by wicking experiments. The surface temperature variation before and after bubble nucleation is in the order of 3 to 4 °C [20], which is similar to the temperature measurement error.

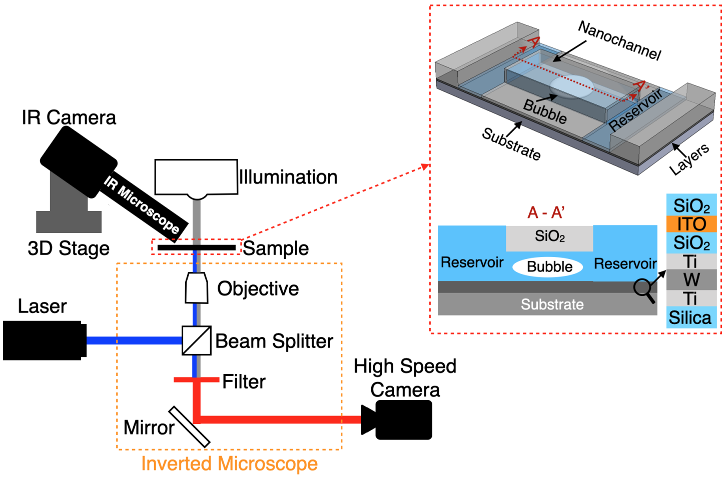

Figure S8: Experimental setup and sample for measurement of nucleation temperature in nanochannels.

Sample for nucleation temperature measurement

The samples for nucleation temperature measurement were fabricated by etching sacrificial chromium (Cr) layer to form the nanochannels [21]. The channels were fabricated on a fused

silica wafer. A 40 nm tungsten (W) film (sandwiched by two 10 nm titanium film as adhesion layer) was firstly deposited. W layer was used to absorb laser power to generate localized heating area. Then, a 300 nm SiO_2 film was deposited using plasma enhanced chemical vapor deposition (PECVD), followed by the physical vapor deposition (PVD) of a 700 nm layer of indium tin oxide (ITO), which is opaque to IR and thus allows for temperature measurement using IR camera. Then, a 1 μm PECVD SiO_2 film was deposited, serving as surface for nanochannel fabrication. A standard lift-off process was used to pattern the sacrificial Cr layer. A 2 μm PECVD SiO_2 film was deposited again to cover the sacrificial Cr layer and was pattern to form the reservoirs. At last, the sacrificial Cr layer was etched using Cr etchant to form the nanochannel.

Temperature measurement

In current work, we use a 700 nm IR opaque ITO layer underneath the nanochannel bottom, to eliminate the noise from the various layers underneath this ITO layer. However, the SiO_2 layer above it still affects temperature measurement. Thus, we calibrated equivalent emissivity of the layers (ITO + SiO_2), using a sample with heating layers on the back (Fig. S9-a). A K-type thermocouple was attached on the back to measure the temperature directly. During the calibration, the surface was heated by passing a direct current in the resistive heating layer on the backside of the sample, and the temperature of the top surface was measured by the IR camera. After reaching steady state, the emissivity of the layers was manually tuned carefully until the the temperature measured by IR camera matches that from thermocouple. The temperature difference between top and bottom surfaces of the sample was ignored as it was in the order of 0.05 °C, resulted from the maximum heating heat flux of $\sim$1.08 W/cm^2. This process was repeated with various sample temperature by varying heating power to obtain the equivalent emissivity at different temperature. As shown in Fig. S9-b, the equivalent emissivity is a constant (0.264 ± 0.008) in the temperature range from 80 °C to 140 °C, similar to reported ITO emissivity in the literature [22, 23, 24], implying weak effect of microscale SiO_2 layer.

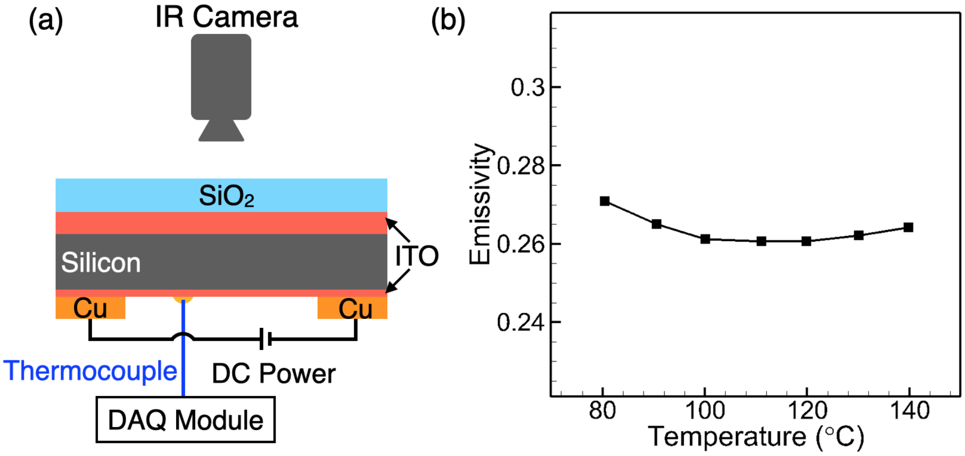

Figure S9: (a) Experimental setup for equivalent emissivity calibration; and (b) the relation between the emissivity and surface temperature.

References

[1] N. R. Tas, J. Haneveld, H. V. Jansen, M. Elwenspoek, and A. van den Berg, "Capillary filling speed of water in nanochannels," *Applied Physics Letters*, vol. 85, no. 15, pp. 3274–3276, 2004.

[2] J. Haneveld, N. R. Tas, N. Brunets, H. V. Jansen, and M. Elwenspoek, “Capillary filling of sub-10nm nanochannels,” *Journal of Applied Physics*, vol. 104, no. 1, p. 014309, 2008.

[3] V. N. Phan, P. Joseph, L. Djeghlaf, A. E. D. Allouch, D. Bourrier, P. Abgrall, A.-M. Gué, C. Yang, and N.-T. Nguyen, “Capillary filling in nanochannels—modeling, fabrication, and experiments,” *Heat Transfer Engineering*, vol. 32, no. 7-8, pp. 624–635, 2011.

[4] M. Yang, B.-Y. Cao, W. Wang, H.-M. Yun, and B.-M. Chen, “Experimental study on capillary filling in nanochannels,” *Chemical Physics Letters*, vol. 662, pp. 137 – 140, 2016.

[5] S. Mozaffari, P. Tchoukov, A. Mozaffari, J. Atias, J. Czarnecki, and N. Nazemifard, “Capillary driven flow in nanochannels – application to heavy oil rheology studies,” *Colloids and Surfaces A: Physicochemical and Engineering Aspects*, vol. 513, pp. 178 – 187, 2017.

[6] V.-N. Phan, C. Yang, and N.-T. Nguyen, “Analysis of capillary filling in nanochannels with electroviscous effects,” *Microfluidics and Nanofluidics*, vol. 7, no. 4, p. 519, 2009.

[7] C. L. Ren and D. Li, “Improved understanding of the effect of electrical double layer on pressure-driven flow in microchannels,” *Analytica Chimica Acta*, vol. 531, no. 1, pp. 15 – 23, 2005.

[8] C. Yang and D. Li, “Electrokinetic effects on pressure-driven liquid flows in rectangular microchannels,” *Journal of Colloid and Interface Science*, vol. 194, no. 1, pp. 95 – 107, 1997.

[9] N. R. Tas, P. Mela, T. Kramer, J. W. Berenschot, and A. van den Berg, “Capillarity induced negative pressure of water plugs in nanochannels,” *Nano Letters*, vol. 3, pp. 1537–1540, 11 2003.

[10] N. R. Tas, M. Escalante, J. W. van Honschoten, H. V. Jansen, and M. Elwenspoek, “Capillary negative pressure measured by nanochannel collapse,” *Langmuir*, vol. 26, pp. 1473–1476, 02 2010.

[11] Z. Ye, S. Li, C. Wang, R. Shen, and W. Wen, “Capillary flow control in nanochannels via hybrid surface,” *RSC Advances*, vol. 6, pp. 2774–2777, 2016.

[12] A. Han, G. Mondin, N. G. Hegelbach, N. F. de Rooij, and U. Staufer, “Filling kinetics of liquids in nanochannels as narrow as 27 nm by capillary force,” *Journal of Colloid and Interface Science*, vol. 293, no. 1, pp. 151 – 157, 2006.

[13] N. A. Mortensen and A. Kristensen, “Electroviscous effects in capillary filling of nanochannels,” *Applied Physics Letters*, vol. 92, no. 6, p. 063110, 2008.

[14] S. Levine, J. R. Marriott, and K. Robinson, “Theory of electrokinetic flow in a narrow parallel-plate channel,” *Journal of the Chemical Society, Faraday Transactions 2: Molecular and Chemical Physics*, vol. 71, pp. 1–11, 1975.

[15] D. J. McGinty, “Molecular dynamics studies of the properties of small clusters of argon atoms,” *The Journal of Chemical Physics*, vol. 58, no. 11, pp. 4733–4742, 1973.

[16] S. Plimpton, “Fast parallel algorithms for short-range molecular dynamics,” *Journal of Computational Physics*, vol. 117, no. 1, pp. 1 – 19, 1995.

[17] S. Nosé, “A unified formulation of the constant temperature molecular dynamics methods,” *The Journal of Chemical Physics*, vol. 81, no. 1, pp. 511–519, 1984.

[18] W. G. Hoover, “Canonical dynamics: Equilibrium phase-space distributions,” *Physical Review A*, vol. 31, pp. 1695–1697, Mar 1985.

[19] S. Poudel, A. Zou, and S. C. Maroo, “Implementation of near-surface disjoining pressure effect in continuum simulations,” *arXiv*, p. 2105.08483, 2021.

[20] C. Gerardi, J. Buongiorno, L. wen Hu, and T. McKrell, “Study of bubble growth in water pool boiling through synchronized, infrared thermometry and high-speed video,” *International Journal of Heat and Mass Transfer*, vol. 53, no. 19, pp. 4185–4192, 2010.

[21] A. Zou, S. Poudel, S. P. Raut, and S. C. Maroo, “Pool boiling coupled with nanoscale evaporation using buried nanochannels,” *Langmuir*, vol. 35, pp. 12689–12693, 10 2019.

[22] K. Sun, W. Zhou, X. Tang, Z. Huang, F. Lou, and D. Zhu, “Effect of the heat treatment on the infrared emissivity of indium tin oxide (ito) films,” *Applied Surface Science*, vol. 257, no. 22, pp. 9639–9642, 2011.

[23] D. Alonso-Álvarez, L. Ferre Llin, A. Mellor, D. J. Paul, and N. J. Ekins-Daukes, “Ito and azo films for low emissivity coatings in hybrid photovoltaic-thermal applications,” *Solar Energy*, vol. 155, pp. 82–92, 2017.

[24] P. Biswas, A. De, N. Pramanik, P. Chakraborty, K. Ortner, V. Hock, and S. Korder, “Effects of tin on ir reflectivity, thermal emissivity, hall mobility and plasma wavelength of sol–gel indium tin oxide films on glass,” *Materials Letters*, vol. 57, no. 15, pp. 2326–2332, 2003.
